# The updated retrospective questionnaire study of sporadic inclusion body myositis in Japan

**DOI:** 10.1186/s13023-019-1122-5

**Published:** 2019-06-26

**Authors:** Naoki Suzuki, Madoka Mori-Yoshimura, Satoshi Yamashita, Satoshi Nakano, Ken-ya Murata, Megumi Mori, Yukie Inamori, Naoko Matsui, En Kimura, Hirofumi Kusaka, Tomoyoshi Kondo, Hidefumi Ito, Itsuro Higuchi, Akihiro Hashiguchi, Hiroyuki Nodera, Ryuji Kaji, Maki Tateyama, Rumiko Izumi, Hiroya Ono, Masaaki Kato, Hitoshi Warita, Toshiaki Takahashi, Ichizo Nishino, Masashi Aoki

**Affiliations:** 1 Department of Neurology, Tohoku University Graduate School of Medicine, 1-1 Seiryo-machi, Aoba-ku, Sendai, 980-8574 Japan; 2 Department of Neurology, National Center Hospital, National Center of Neurology and Psychiatry (NCNP), Tokyo, Japan; 3 Department of Neurology, Graduate School of Medical Sciences, Kumamoto University, Kumamoto, Japan; 4 Department of Neurology, Osaka City General Hospital, Osaka, Japan; 5 Department of Neurology, Wakayama Medical University, Wakayama, Japan; 6 Department of Neurology and Geriatrics, Kagoshima University Graduate School of Medical and Dental Sciences, Kagoshima, Japan; 7 Department of Clinical Neuroscience, Institute of Biomedical Sciences, Tokushima University Graduate School, Tokushima, Japan; 8 Department of Neurology, Kansai Medical University, Osaka, Japan; 9 Department of Neurology, National Hospital Organization Iwate National Hospital, Iwate, Japan; 10 Department of Neurology, Southern Tohoku General Hospital, Iwanuma, Miyagi Japan; 11 Department of Neurology, Sendai-Nishitaga National Hospital, Sendai, Japan; 12 Department of Neuromuscular Research, National Institute of Neuroscience and Department of Genome Medicine Development, Medical Genome Center, National Center of Neurology and Psychiatry (NCNP), Tokyo, Japan

**Keywords:** Sporadic inclusion body myositis, Multicenter survey, Questionnaire, Aging, Muscle diseasef

## Abstract

**Background:**

Sporadic inclusion body myositis (sIBM) is the most prevalent muscle disease in elderly people, affecting the daily activities. sIBM is progressive with unknown cause and without effective treatment. In 2015, sIBM was classified as an intractable disease by the Japanese government, and the treatment cost was partly covered by the government. This study aimed to examine the changes in the number of patients with sIBM over the last 10 years and to elucidate the cross-sectional profile of Japanese patients with sIBM.

**Methods:**

The number of sIBM patients was estimated through a reply-paid postcard questionnaire for attending physicians. Only patients diagnosed as “definite” or “probable” sIBM by clinical and biopsy sIBM criteria were included in this study (Lancet Neurol 6:620-631, 2007, Neuromuscul Disord 23:1044-1055, 2013). Additionally, a registered self-administered questionnaire was also sent to 106 patients who agreed to reply via their attending physician, between November 2016 and March 2017.

**Results:**

The number of patients diagnosed with sIBM for each 5-year period was 286 and 384 in 2011 and 2016, respectively. Inability to stand-up, cane-dependent gait, inability to open a plastic bottle, choking on food ingestion, and being wheelchair-bound should be included as sIBM milestones. Eight patients were positive for anti-hepatitis C virus antibody; three of them were administered interferon before sIBM onset. Steroids were administered to 33 patients (31.1%) and intravenous immunoglobulin to 46 patients (43.4%). From 2016 to 2017, total of 70 patients applied for the designated incurable disease medical expenses subsidy program. Although the treatment cost was partly covered by the government, many patients expressed psychological/mental and financial anxieties.

**Conclusions:**

We determined the cross-sectional profile of Japanese patients with sIBM. Continuous support and prospective surveys are warranted.

## Background

Sporadic inclusion body myositis (sIBM) is the most frequent inflammatory muscle disease in middle-aged and elderly people [3, 10]. sIBM symptoms typically include muscle weakness/atrophy in the quadriceps, wrist, and finger flexors as well as dysphagia. Muscle biopsy typically reveals endomysial inflammation, mononuclear cell invasion into non-necrotic fibers, and rimmed vacuoles, suggesting inflammation and degeneration as the underlying pathological mechanisms. The effects of immunological treatment such as steroid administration are limited [14]. Thus, a treatment with bimagrumab, an activin receptor antagonist, was developed [1], but was discontinued in April 2016.

We previously conducted a retrospective survey of Japanese patients with sIBM at the National Center of Neurology and Psychiatry (NCNP). Although physicians’ awareness of sIBM after the 1970s led to a detection bias, the increasing incidence of sIBM in Japan ensued after a rapid change in dietary habits from a traditional to a Westernized diet post-World War II, suggesting that diet may influence the incidence of sIBM in Japan [17]. Another group has also reported that the number of Japanese patients with sIBM increased in recent years [11].

This study aimed to examine the changes in the number of patients with sIBM over the last 10 years. Additionally, a retrospective cross-sectional analysis of the status of sIBM therapy in Japan was performed.

## Materials and methods

### Reply-paid postcard questionnaire for attending physicians

Reply-paid postcard questionnaires were sent to the board-certified members of the Japanese Society of Neurology. In our previous study, the number such questionnaires sent was 4857 [18], whereas in the present study, 5500 were sent. The contents of the questionnaire are listed in Table 1. In the previous study, the number of newly diagnosed patients between 2005 and 2009 was determined, whereas in the present study, that between 2011 and 2015 was determined. Additionally, the attending physicians were asked to request their patients to provide detailed answers to the contents of the questionnaire. Only patients with “definite” or “probable” sIBM detected based on clinical and biopsy criteria were included in the study [12, 13].

**Table 1:** Reply-paid postcard questionnaire for neurologists

Basic information	Hospital
	Doctor’s name
	Address/Phone/E-mail
Newly diagnosed	Number of patients in 5 years
	2005–2009 or 2011–2015
Able to ask patients for questionnaire in detail	Yes/No

### Detailed questionnaire for patients and caregivers

**Fig. 1:**
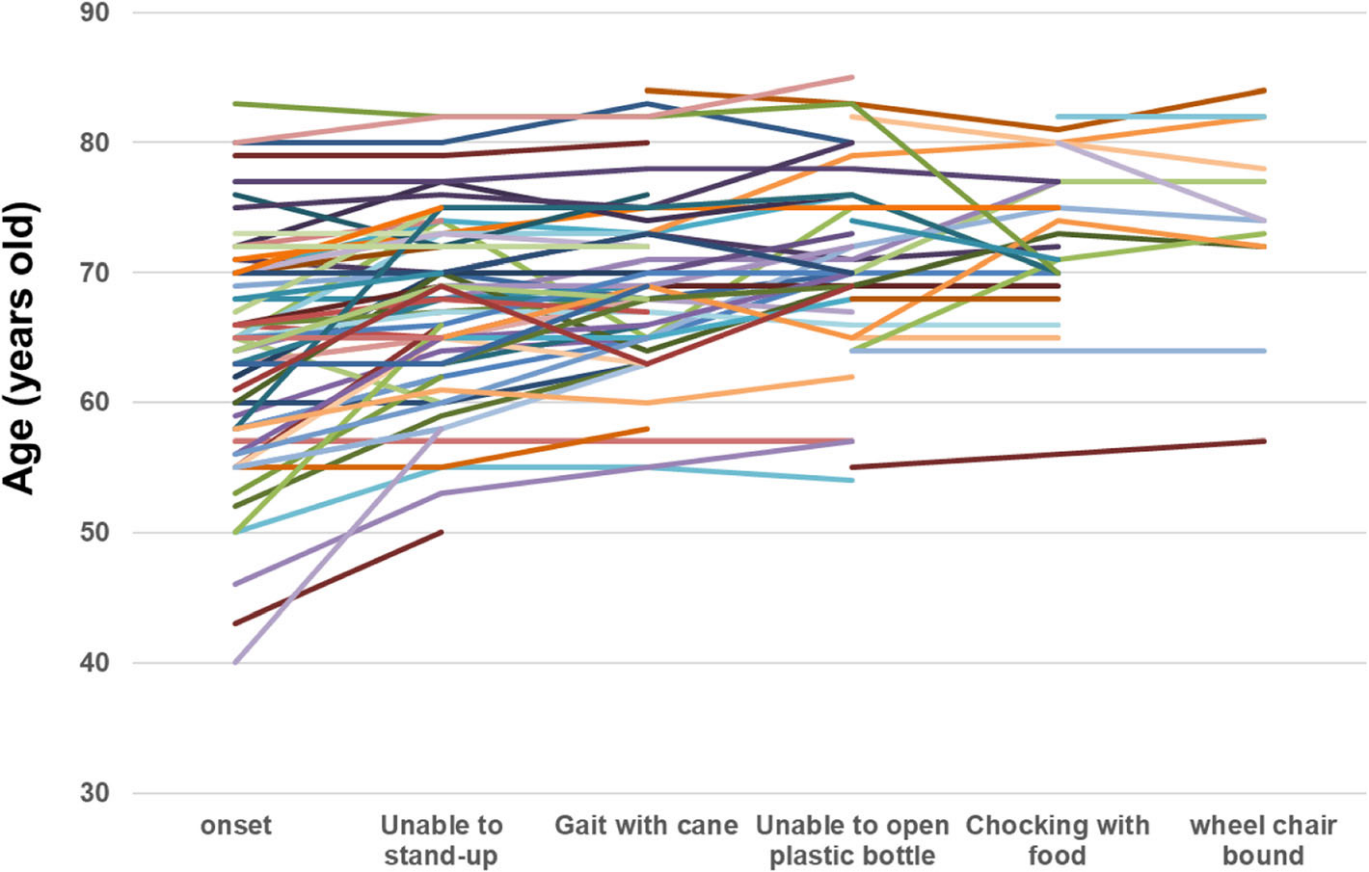
Transitive change of important milestones in individual cases of sIBM

Between November 2016 and March 2017, a registered self-administered questionnaire, with an explanation of the study’s purpose, was distributed to 106 patients who had agreed to reply via their attending physicians. Participation in the study was emphasized to be completely voluntary. To ensure confidentiality, patients returned the questionnaires in envelopes they had sealed themselves. The questionnaire included contents pertaining to past medical history, complications, family medical history, sIBM onset, ambulation status, and with/without muscle biopsy. It also included information on name, age, height, weight, lifestyle, economic status, psychological stress. The structure of the questionnaire for patients and caregivers is presented in Table 2. To determine the trend over time, several milestones were plotted in one graph for all patients in Fig. 1.

**Table 2:** List of questions for the patients and caregivers

Basic information	Hospital
	Date
	Doctor’s name
	Patient’s Name
	Date of birth and age
	Sex
	Address/Phone/E-mail
Life/Past History	Development
	Exercise Capacity at School
	Works
	Preference of food
	Economic matters
Symptoms	Initial symptom: unable to stand-up etc.
	Milestones: wheelchair, cane, dysphagia
	Mental/psychological stress
Diagnosis	Age at admission
	Method of diagnosis: muscle biopsy
	Family history, Past history
Therapy	Rehabilitation
	Steroid/IVIG/others
	Interferon
For Caregiver	Activities of daily life
	Mental/psychological stress
	Cognitive decline: none, age-appropriate, diagnosed as dementia, medication
Functional Scale	Modified IBMFRS

### Ethics approval and consent to participate

The study protocol was approved by the Ethics Committee of Tohoku University School of Medicine. Informed consent was obtained from each of the participants after the purpose of the study had been explained to them. Participants were allowed to decide whether or not to participate in the study.

### Modified inclusion body myositis-functional rating scale (IBMFRS)

To clinically predict the course of the sIBM, we investigated whether its severity was related to other parameters. We referred to IBMFRS [5, 8] and asked the patients to rate their status following the grading scale shown in Table 3. We also examined whether some quantifiable parameters were related to sIBM severity.

**Table 3:** Modified IBM Functional Rating Scale (IBMFRS)

1. Swallowing	5 Dressing	9 Walking
4 Normal	4 Normal	4 Normal
3 Early eating problems occasional choking	3 Independent but with increased effort or decreased efficiency.	3 Slow or mild unsteadiness
2 Dietary consistency changes	2 Independent but requires assistive devices or modified techniques	2 Intermittent use of an assistive device (AFO, cane walker)
1 Frequent choking	1 Requires assistance from caregiver for some clothing items (Velcro, snaps,shirts,shirts without buttons, etc.)	1 Unable to walk without assistive device
0 Needs tube feeding	0 Total dependence	0 Wheelchair dependent
2 Handwriting (with dominant hand prior to IBM onset)	6 Hygiene (Bathing & Toileting)	10 Climbing Stairs
4 Normal	4 Normal	4 Normal
3 Slow or sloppy; all words are legible	3 Independent but with increased effort or decreased activity	3 Slow with hesitation or increased effort; uses hand rail intermittently
2 Not all words are legible	2 Independent but requires use of assistive devices (shower chair, raised toilet seat, etc)	2 Dependent on hand rail
1 Able to grip pen but unable to write	1 Requires occasional assistance from caregiver	1 Dependent on hand rail and additional support (cane or person)
0 Unable to grip pen	0 Completely dependent	0 Cannot climb stairs
3 Cutting Food & Handling Utensils	7 Turning In Bed & Adjusting Covers	
4 Normal	4 Normal	
3 Somewhat slow and clumsy, but no help needed	3 Somewhat slow and clumsy but no help needed	
2 Can cut most foods although clumsy and slow; some help needed; can’t use chopsticks	2 Can turn alone or adjust sheets, but with great difficulty	
1 Food must be cut by someone, but can still feed slowly	1 Can initiate, but not turn or adjust sheets alone (needs caregivers)	
0 Needs to be fed	0 Unable or requires total assistance	
4 Fine Motor Tasks (Opening doors, using keys & picking up small objects)	8 Sit to Stand	
4 Independent	4 Independent (without use of arms)	
3 Slow or clumsy in completing task	3 Performs with substitute motions (leaning forward, rocking) but without use of arms	
2 Independent but requires modified techniques or assistive devices	2 Requires use of arms	
1 Frequently requires assistance from caregiver (e.g. bottons)	1 Requires assistance from a device or person	
0 Unable	0 Unable to stand	

### Data analysis

Data were summarized using descriptive statistics, including mean, standard deviation (SD), median, range, frequency, and percentage. Statistical analysis was performed using Pearson’s chi-square test and Log-rank test for Kaplan-Meier analysis with JMP Pro software (ver. 14.0.0).

## Results

In our previous study, we sent a total of 4857 questionnaires [18], whereas in the present study, 5500 questionnaires were sent. The number of responses were 1253 and 1316 in the previous and present studies (Table 4), indicating a reply rate of 25.8 and 23.9%, respectively. The number of patients diagnosed with sIBM for each 5-year period was 286 and 384, respectively (Table 4).

**Table 4:** Diagnosed sIBM patients / 5 years from reply-paid postcard Questionnaire

Term (year)	Sent Cards	Reply	Reply ratio (%)	Diagnosed patients / 5 years
2005–2009	4857	1253	25.8	286
2011–2015	5500	1316	23.9	384

To further elucidate the course of sIBM, a detailed version of the questionnaire for was sent to both patients and caregivers. Within our cohort, male patients were more prevalent than females (males: *n* = 77; females: *n* = 29). The mean age at sIBM onset was 62.15 ± 9.25 (median, 63; range, 40–84) years. During the present study, the average time from sIBM onset to questionnaire administration was 9.37 years (median, 7; SD = 6.89). Additionally, the respondents were asked to mention important disease milestones (Table 5). In the previous study, the most common initial symptom was the weakness of the proximal lower muscles, including the quadriceps femoris (*n* = 117, 80%), followed by the weakness of finger flexors (*n* = 9) and shoulder girdle muscle (*n* = 5), muscle pain (*n* = 3), general fatigue (*n* = 3), and dysphagia (*n* = 5) [18]. In the present study, we aimed to identify an array of milestones based on patients’ reports. The inability to stand-up occurred at the age of 66.38 (described in 86 patients, SD = 7.74). Cane-dependent gait occurred at the age of 69.08 years (*n* = 67), followed by the inability to open a plastic bottle at 70.3 years (*n* = 53), choking on food at 71.17 years (*n* = 36), and becoming wheelchair-bound at 71.64 years (*n* = 33).

**Table 5:** Milestones from the questionnaire study

	Onset	Unable to stand-up	Gait with cane	Unable to open plastic bottle	Choking with food	wheel chair bound
Average (y.o.)	62.15	66.38	69.08	70.3	71.17	71.64
SD (years)	9.25	7.74	6.9	7.16	6.28	6.86
n	106	86	67	53	36	33

**Fig. 2:**
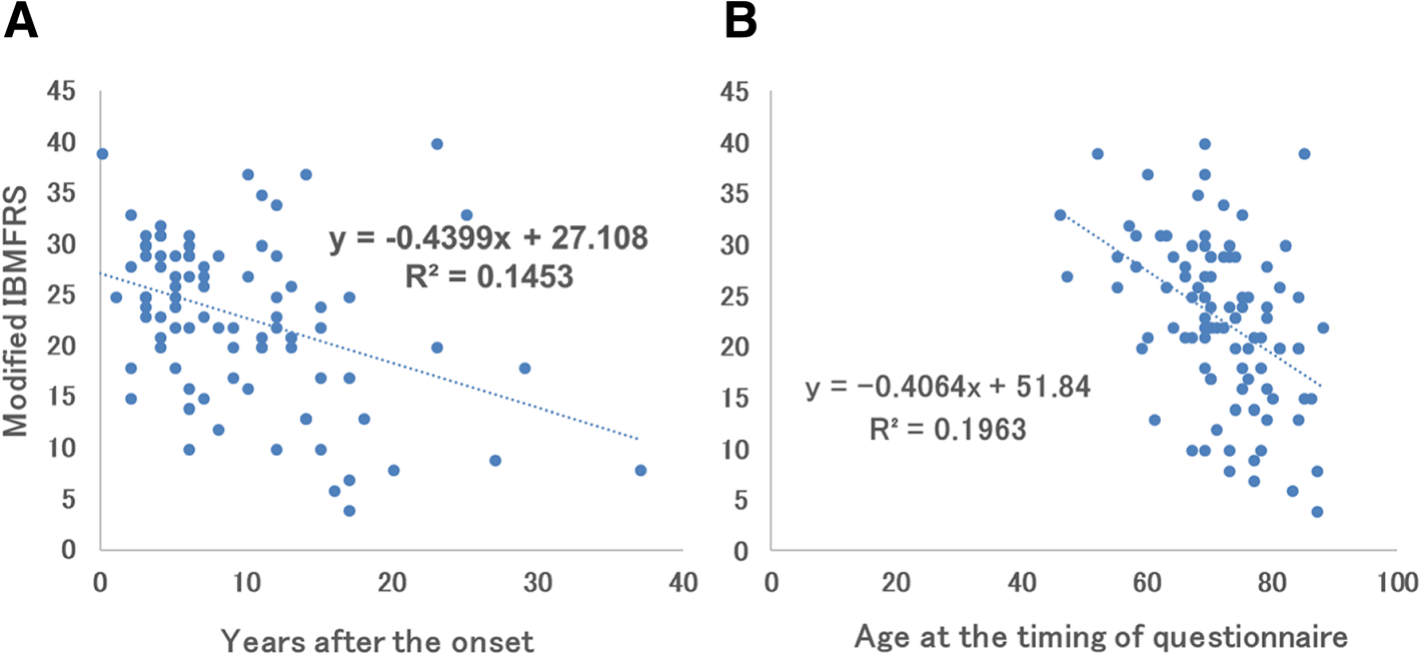
**a** The correlation coefficient between years after the onset and modified IBMFRS is 0.1453. **b** The correlation coefficient between age at the timing of the questionnaire and modified IBMFRS is 0.1963

One milestone was plotted in one graph for all patients (Fig. 1). Next, we examined whether some quantifiable parameters were related to sIBM severity. The correlation coefficient between the time after sIBM onset and modified IBMFRS was 0.1453 (Fig. 2a). Moreover, the correlation coefficient between age at the time of the study and modified IBMFRS was 0.1963 (Fig. 2b). No correlation was found between modified IBMFRS and age at sIBM onset or Brinkman index (data not shown).

**Fig. 3:**
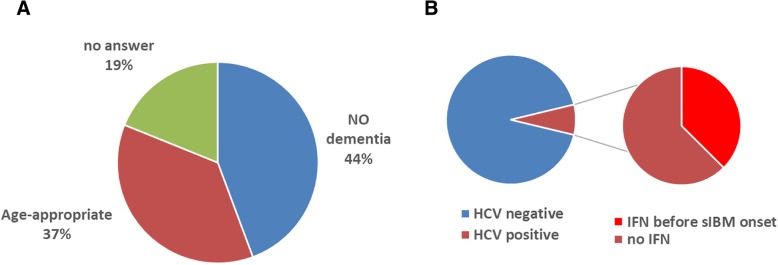
**a** Subjective assessment of dementia among sIBM cases by caregivers. **b** Eight patients (7.5%) described that they are HCV positive. Three of them underwent the administration of interferon for the therapeutic purpose of HCV.

The 106 patients in this study did not exhibit signs of cognitive impairment, as assessed by the caregivers (Fig. 3a) with the questionnaire of “Cognitive decline: none, age-appropriate, diagnosed as dementia, medication”. Hepatitis C virus (HCV) infection has been discussed in the context of sIBM pathogenesis [21]. In the present study, eight patients (7.5%) were HCV positive, and three of them received interferon treatment before sIBM onset. HTLV1 was not mentioned by the patients in the present study.

From a therapeutic point of view, 67 patients underwent an sIBM rehabilitation program. Steroids were administered to 33 patients (31.1%), whereas 46 (43.4%) received intravenous immunoglobulin (IVIG), 2 received immunosuppressants, and 21 received both. Sixteen (48.5%) and 20 (43.5%) patients who were administered steroids and IVIG at least one time during the disease course, respectively, subjectively reported some improvement. Four patients opted for tube feeding or gastrostomy.

**Fig. 4:**
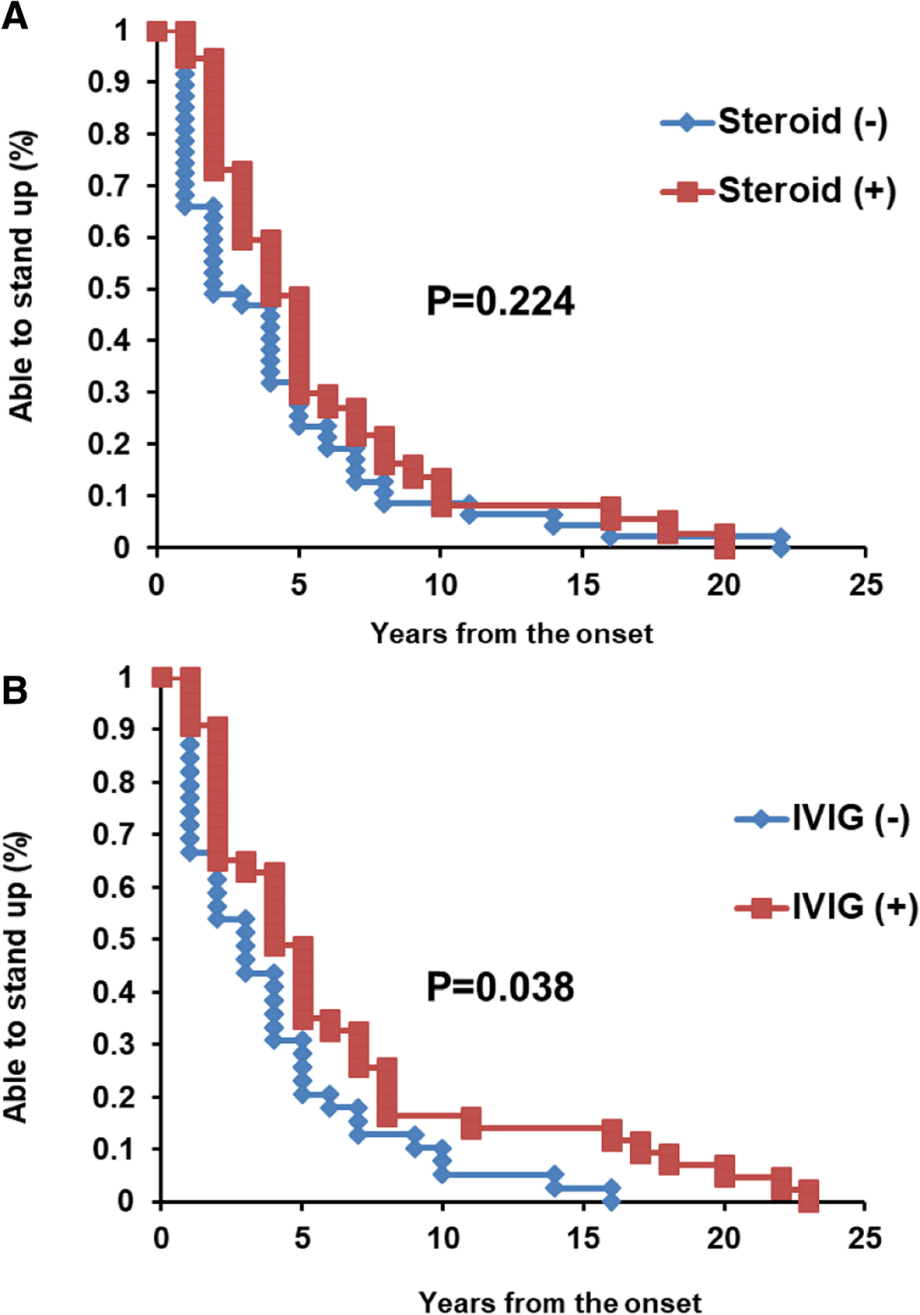
Interventions of this cohort in Multicenter survey. **a** No significant difference was found in the term of unable to stand-up from the onset between the group with or without steroid (*p* = 0.224: Log-rank test.). **b** IVIG administered patients showed significantly longer term of stand-up by themselves (*p* = 0.038; Log-rank test.).

We investigated whether these interventions modified the course of sIBM. Given the limited milestone description and number of patients, the inability to stand-up was selected as the index of sIBM progression. No significant difference was observed with regard to steroid administration (*p* = 0.224, Fig. 4a). However, patients administered with IVIG could independently stand-up for a longer period of time than patients without IVIG (*p* = 0.038, Fig. 4b). IVIG or steroid administration didn’t affect the timing of wheelchair bound (*p* = 0.558 and 0.856, data not shown). The correlation between IVIG administration and age at onset (*p* = 0.2931), sex (*p* = 0.9835), age at the questionnaire (*p* = 0.5306), whether unable to stand-up (*p* = 0.8380) were not significant (Pearson’s chi-square test).

In 2015, sIBM was classified as an intractable disease by the Japanese government. The treatment cost was partly covered by the government. From 2016 to 2017, 70 patients applied for the designated incurable disease medical expenses subsidy program. However, 47 patients (44.3%) still experienced psychological/mental and financial anxieties.

## Discussion

The present study describes the results of a reply-paid postcard questionnaire survey directed at attending physicians. One limitation is any genetic testing was not mandatory for the inclusion criteria of this study. We selected the patients fulfilling the “definite” or “probable” sIBM criteria by clinical and biopsy (Ref.) which reduce the possibility of including myofibrillar myopathy, GNE myopathy or VCP myopathy. Reply rate is around 25% for the reply-paid postcard questionnaire for neurologists. In Japan, diagnosis of the sIBM patients would be examined mostly at either the National Center or University Hospitals. Although the percentage is small, most of the physicians who underwent muscle biopsy at university or central regional hospitals might reply and non-responders might not see the patients. Over the last 10 years, we repeatedly performed a nationwide survey. We found the number of patients with sIBM is increasing in Japan (Table 4), particularly increasing linearly among individuals born after the 1920s. Previously, we performed a retrospective survey involving Japanese patients with sIBM who were diagnosed at the NCNP [17]. Moreover, another research group from Japan reported an increasing number of patients sIBM [11]. One possible reason for this trend is the increasing awareness of sIBM among medical doctors. Doctors of other specialties, such as orthopedic surgeons or otolaryngologists, can also diagnose sIBM, and its prevalence is likely to increase among elderly people in the near future. Inability to stand-up, cane-dependent gait, inability to open a plastic bottle, choking on food, and being wheelchair-bound are significant sIBM milestones. In the disease course of sIBM, patient sometimes can’t stand-up by themselves because of the weakness of quadriceps muscle, but can walk with cane. This increasing trend is easily observed as the patient aged. Aspiration pneumonia and being wheelchair-bound occurred approximately 10 years after sIBM onset (Table 5 and Fig. 1). These milestones are similar to those previously reported [2, 6, 7], and can help inform the patients about the disease. In outpatient clinics, improving mobility using a walking device or chair should be emphasized for the first 5 years after sIBM onset. On the other hand, paying greater attention to dysphagia and wheelchair requirement should be the focus of the latter 5 years. Additionally, a fraction of the patients were observed to initially have partial or single sIBM symptom (e.g., dysphagia or inability to flex fingers), which may remain isolated for several years [15, 16]. No correlation was found between modified IBMFRS and the parameters examined (data not shown). These facts indicate that sIBM is a heterogeneous disease. Since the questionnaire survey was conducted in a cross-sectional manner, a time-course study should be planned to further investigate this correlation. The age at the time of the study was negatively, but very weakly, correlated with modified IBMFRS, suggesting that aged patient manifest impairments in various activities of daily living. Follow-up time course analysis is desirable in the future study [5].

In the present study, none of the patients exhibited signs of apparent dementia (Fig. 3a), as subjectively evaluated. This is consistent with the findings of our previous survey [18]. Further structured questionnaire in detail should be examined to analyze the severity of dementia. On the other hand, inclusion body myopathy with Paget’s disease of the bone and frontotemporal dementia or multisystem proteinopathy coexisted with dementia [20, 22], indicating that sIBM should be separated from diseases associated with genetic mutations.

Eight patients (7.5%) were anti-HCV antibody positive, and three underwent interferon treatment before sIBM onset. The prevalence of HCV was estimated around 2% over 70 years of age in Japan [19]. Compared to the national scale data, the prevalence ratio of HCV antibody positive patients seemed to be rather high. This suggests that for treating sIBM, information pertaining to the viral infection and immune modulation therapy should be collected.

“Can rehabilitation slow sIBM progression?” is a frequently asked clinical question. In the present study, we observed no significant differences between patients with and without involvement in sIBM rehabilitation programs in terms of the course of sIBM, as evaluated by the time required to exhibit inability to stand-up. A recent study reported that 12 weeks of low-load, blood flow-restricted, resistance training did not improve self-reported or objective physical function among patients with sIBM [9]. The authors claimed that the training protocol had a preventive (retaining) effect on the sIBM-related decline in leg muscle strength, which may aid the long-term preservation of physical function and postpone the need for healthcare assistance, and maintain the ADLs. The Hybrid Assistive Limb has been approved for sIBM rehabilitation in Japan. However, prospective evaluations with structured questionnaire and clinical trial are necessary to validate this new therapeutic strategy.

Our results indicate subjective symptom improvement through immune-mediated therapies. We also found that patients in the IVIG-treated cohort required longer time to exhibit the inability to stand-up (Fig. 4b). However, there are several limitations in this result. Since this was a retrospective analysis, there were both selection and observational biases. The timing and term of administration were not unified. The small number of patients with the milestone of the wheelchair bound might affect the result of the no correlation between therapy and wheelchair bound. Analysis of larger number of patients with prospective unified protocol is mandatory.

In a previous study, IVIG improved four cases of sIBM in terms of dysphagia in 8 months [4]. Although the effect of IVIG does not last long, in Australia, patients with sIBM with severe dysphagia are covered by insurance [10]. Benveniste et al. reported that 71 (52%) patients received immunosuppressive treatments such as prednisolone (91.5%) or other immunomodulatory drugs, including IVIG, methotrexate, or azathioprine (64.8%), for a median duration of 40.8 months. The heterogeneity of sIBM might mask the effect of drugs such as bimagrumab, leading to clinical trial termination. For the slowly progressive neuromuscular disease like sIBM, it would be practical to monitor only a small number of evaluation item (e.g. unable to stand-up) and follow-up for longer period.

Developed countries such as Japan have an aged population, and mid- to older-aged partners of patients with sIBM often lack physical strength and may also have a disease of their own. Our previous questionnaire also revealed several qualitative aspects pertaining to caregivers, typically spouses, and their difficulty in managing sIBM, given its long course. In this study, 70 patients applied for the designated incurable disease medical expenses subsidy program by Japanese government. Clearly, this has an impact on caregivers who themselves require societal supports. However, 47 patients (44.3%) still reported psychological/mental and financial anxieties.

The present study has several limitations, as previously mentioned. The study used a retrospective and cross-sectional design and, thus, could not determine causal relationships. A longitudinal study should be conducted to address this issue.

## Conclusions

Our multicenter patient and caregiver questionnaire survey revealed that the phenotypes of Japanese patients with sIBM are similar to those of Western country patients with sIBM, at least through a cross-sectional methodology. Many patients described psychological/mental and financial anxiety, given their old age. Thus, a follow-up survey is warranted to determine the prospective natural history of sIBM in Japan.

## Data Availability

An outline of the questionnaire used for this study is available in Table 3. Please contact author for data request.

## Abbreviations

FDP: Flexor digitorum profundus
IBMFRS: IBM functional rating scale
IBMPFD: Inclusion body myopathy with Paget’s disease of the bone and fronto-temporal dementia
IFN: Interferon
IVIG: Intravenous immunoglobulin
MSP: Multisystem proteinopathy
PSL: Prednisolone
sIBM: Sporadic inclusion body myositis
